# Evaluation of Strategies to Produce Highly Porous Cross-Linked Aggregates of Porcine Pancreas Lipase with Magnetic Properties

**DOI:** 10.3390/molecules23112993

**Published:** 2018-11-16

**Authors:** José Renato Guimarães, Raquel de Lima Camargo Giordano, Roberto Fernandez-Lafuente, Paulo Waldir Tardioli

**Affiliations:** 1 Graduate Program in Chemical Engineering, Department of Chemical Engineering, Federal University of São Carlos, Rod. Washington Luís, km 235, SP-310, São Carlos 13565-905, Brazil; renatoge74@gmail.com (J.R.G.); raquel@ufscar.br (R.d.L.C.G.); 2 Departmento de Biocatálisis, ICP-CSIC, Campus UAM-CSIC Madrid, 28049 Madrid, Spain

**Keywords:** silica magnetic nanoparticles, bovine serum albumin, soy protein, starch, protein surface modifiers

## Abstract

The preparation of highly porous magnetic crosslinked aggregates (pm-CLEA) of porcine pancreas lipase (PPL) is reported. Some strategies to improve the volumetric activity of the immobilized biocatalyst were evaluated, such as treatment of PPL with enzyme surface-modifying agents (polyethyleneimine or dodecyl aldehyde), co-aggregation with protein co-feeders (bovine serum albumin and/or soy protein), use of silica magnetic nanoparticles functionalized with amino groups (SMNPs) as separation aid, and starch as pore-making agent. The combination of enzyme surface modification with dodecyl aldehyde, co-aggregation with SMNPs and soy protein, in the presence of 0.8% starch (followed by hydrolysis of the starch with α-amylase), yielded CLEAs expressing high activity (immobilization yield around 100% and recovered activity around 80%), high effectiveness factor (approximately 65% of the equivalent free enzyme activity) and high stability at 40 °C and pH 8.0, i.e., PPL CLEAs co-aggregated with SMNPs/bovine serum albumin or SMNPs/soy protein retained 80% and 50% activity after 10 h incubation, respectively, while free PPL was fully inactivated after 2 h. Besides, highly porous magnetic CLEAs co-aggregated with soy protein and magnetic nanoparticles (pm-SP-CLEAs) showed good performance and reusability in the hydrolysis of tributyrin for five 4h-batches.

## 1. Introduction

Lipases (triacylglycerol acylhydrolase, EC 3.1.1.3) are enzymes whose natural function is the hydrolysis of triglycerides at the water-lipid interface releasing free fatty acids, diglycerides, monoglycerides and glycerol [1,2,3,4]. However, in vitro, they are also able to catalyze esterification and transesterification reactions (acidolysis, alcoholysis and interesterification) in organic media (with restricted water content) [1,2,3,4,5]. This wide lipase specificity, added to their excellent chemo-, regio- and enantioselectivities and/or specificities, have been exploited for several important biotechnological applications in the pharmaceutical, food and agrochemical industries [6,7,8,9,10,11,12]. Among lipases, porcine pancreas lipase (PPL) is widely used in biotransformation reactions in organic media for several industrial applications because of its high selectivity, high solvent tolerance, high catalytic activity, and thermal stability at high temperatures under low water concentrations [8,13,14,15,16].

However, the use of enzymes in their soluble form for large-scale industrial processes is not very attractive, because of their high production cost and low operational stability [17]. A strategy that has been widely exploited to overcome these drawbacks is their immobilization on solid supports, which, if properly performed, can provide several advantages from an industrial point of view, such as enabling continuous and batch processing, improved stability compared to the free enzyme, increased volumetric activity, easy recovery from product stream, and reuse for several cycles [17,18,19,20,21,22].

There are a great number of techniques and supports suitable for enzyme immobilization. In general, some criteria are followed in their choice regarding to high activity of the immobilized enzyme, high stability against temperature and organic solvents, low cost of immobilization, and low toxicity of the immobilization reagents and supports [17,18,23,24,25].

In the case of lipases, a popular technique that has been widely reported is their immobilization by hydrophobic adsorption on highly hydrophobic surfaces [26,27,28,29,30,31,32]. This is because the lipases have a peculiar mechanism, called interfacial activation, a phenomenon that allows the enzyme to exist in two forms in equilibrium: a closed form, in which a lid (polypeptide chain) covers the enzyme active site, and an open form, in which the lid is moved away allowing the lipase to adsorb to hydrophobic surfaces (e.g., drops of oils, air bubbles, etc.) [33,34,35,36] and turning the active site accessible to the substrate [36]. The immobilization on these supports involves and stabilizes the open form of the lipase [37]. Despite the excellent results of this immobilization approach, the use of pre-existing supports increases the final cost of the biocatalyst. Saving biocatalyst cost can be an interesting approach to turn the applied utilization of immobilized lipases still more attractive. In this sense, Sheldon has reported a free-carrier technique for immobilizing enzymes, the crosslinked enzyme aggregates (CLEA) [38]. It allows using non-purified enzyme or even co-immobilizing different enzymes [38,39]. However, it also presents some problems, such as low operational mechanical resistance of the biocatalyst, difficulty of particle recovery, and high intra-particle diffusion limitations [20,40].

The immobilization of enzymes as CLEAs is a simple technique. In a first step, the enzyme is aggregated and precipitated by a precipitant (salts, water-miscible organic solvents, non-ionic polymers, etc.), and in a second step the aggregates are crosslinked with bifunctional (e.g., glutaraldehyde) or multifunctional (e.g., polyaldehyde dextran) agents usually via the amino groups on the enzyme surface [12,41,42,43,44]. When the enzyme surface has a low content of amino groups, co-feeders are commonly used to aid the crosslinking, such as proteins (bovine serum albumin, soy protein, etc.) [15,45,46,47,48,49], polyethyleneimine [50,51], and so on. CLEAs of several enzymes have been reported [12,15,47,52,53,54,55], including bovine and porcine pancreas lipases. Cui et al. [56] reported the co-aggregation of bovine pancreas lipase with bovine serum albumin (BSA, 0.05 g L^−1^) as co-feeder, followed by crosslinking with 1% *w*/*w* glutaraldehyde, reaching 75% catalytic retention. Ramos et al. [15] reported the immobilization of porcine pancreas lipase as CLEAs using ethanol as precipitant (enzyme solution: ethanol volume ratio of 1:3), soy protein as co-feeder (enzyme protein:co-feeder mass ratio of 1:3), and glutaraldehyde as cross-linker (5 μmol of glutaraldehyde groups/mg total protein). These conditions allowed reaching an immobilization yield around 60% and an activity recovery of around 40%.

An important drawback to be considered in the use of CLEAs is the mass transfer limitation within their highly compact supramolecular structure [56]. Some works have reported some modifications of the original method aiming to reduce intra-particle mass transfer limitations, such as the use of starch as pore-making agent. Wang et al. [57] developed a new strategy for the preparation of porous CLEAs (p-CLEAs), which involves co-precipitation of the enzyme with starch and further crosslinking, followed by hydrolysis of the polysaccharide from the aggregate structure by α-amylase. The authors reported that p-CLEAs of papain prepared in this way had larger pore sizes (enabling to reduce mass transport limitation) and large surface compared to the conventional CLEAs. Besides that, p-CLEAs of papain prepared with protein feeders (ovalbumin and BSA) and starch (0.3–0.4%, *w*/*v*) yielded 100% catalytic potential, demonstrating the feasibility of this CLEA preparation method. Recently, Cui et al. [58] reported a new procedure to prepare CLEAs of pancreas lipases in hydrophobic ionic liquid microemulsion aiming to reduce diffusion problems within the CLEA structures. Under optimized conditions, the authors reported the preparation of spherical structures with good dispersity and greater recovery activity (84.6%) compared to the amorphous structure of conventional CLEAs (52.8%).

Other problem of CLEAs is the difficulty of their handling [20], problem that may be solved co-aggregating enzymes and magnetic nanoparticles to produce a magnetic CLEA [59,60,61,62]. This may have also other positive effects on the CLEA (porosity, stability) but involve the use of a “support”.

In this context, this work evaluated some strategies to prepare porous magnetic CLEAs of PPL, aiming to produce biocatalysts with decreased mass transfer limitation, ease of recovery without separation by centrifugation, and increased thermal and mechanical stabilities. A set of experiments was carried out to choose the protein co-feeders, mass ratio between magnetic nanoparticles and protein co-feeder, concentration of starch as pore making agent, and modification of the enzyme surface using polyethyleneimine or hydrophobic polyaldehydes. For the best PPL CLEAs, their activities and thermal stabilities, performance in the tributyrin hydrolysis, and reusability were characterized.

## 2. Results and Discussion

### 2.1. Influence of Co-Feeders in the Preparation of CLEAs

Bovine serum albumin (BSA) and soy protein (SP) were evaluated as co-feeders, and silica magnetic nanoparticles functionalized with amino groups (SMNPs) were employed to produce magnetic CLEAs. Table 1 shows that when using protein co-feeders (BSA or SP), the recovered activities of CLEAs were around 6.5 and 15.7 times higher than when prepared without any co-feeder. Although the co-feeders allowed the preparation of more active CLEAs, the immobilization yield in the presence of SP was reduced from around 65% to 51%, while in the presence of BSA the immobilization yield was increased from around 65% to 94%.

**Figure 1:**
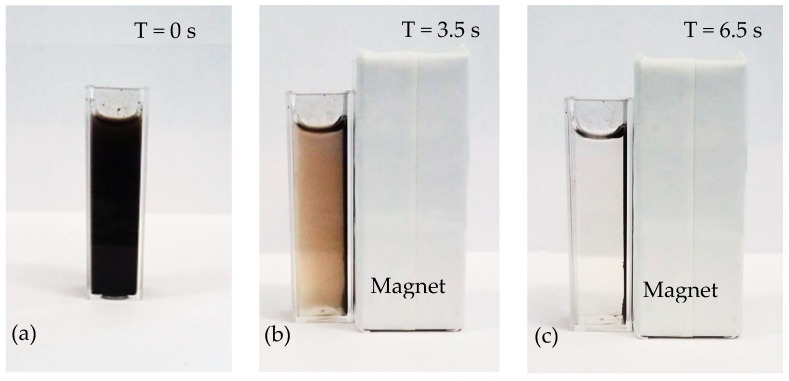
Images of magnetic CLEA separation by applying an external magnetic field: (**a**) CLEA suspension, (**b**) magnetic field capturing the CLEA, and (**c**) CLEA separated from the suspension.

CLEAs prepared with SMMPs also showed an increase in the recovered activity (8.4 times), with no significant decrease in the immobilization yield (from 65% to 63%). However, a combination of protein co-feeders and SMNPs (mass ratio of 1:1) showed to be the best strategy, particularly in the case of BSA/SMMPs, which yielded CLEAs with immobilization yield around 70% and recovered activity of 25.43% (around 17 times higher than that in the absence of co-feeders (where recovered activity was only 1.46%)). Besides the improvement in the recovered activity, these CLEAs have magnetic properties, which allow their easy recovery applying an external magnetic field (Figure 1).

Several studies have showed that CLEAs co-aggregated with protein co-feeders (BSA or SP) exhibit better performance regarding to enzyme leaching during washing steps and reduced mass transfer limitations during the reaction, mainly using macromolecular substrates [15,45,54,63]. These better performances are related to the surface amino groups from lysine residues present in the structure of these protein co-feeders, aiding the establishment of covalent bonds between the enzyme and the feeder forming the CLEA matrix, thus reducing enzyme leaching [45,53,63,64]. It is noteworthy that SMNPs also provided to the CLEAs these improvements, since they are functionalized with primary amino groups derived from the functionalization reagent (3-aminopropyltriethoxysilane). Besides, the use of co-feeders can reduce or avoid diffusion problems by diluting the active enzyme, increasing the recovered activity [47].

**Figure 2:**
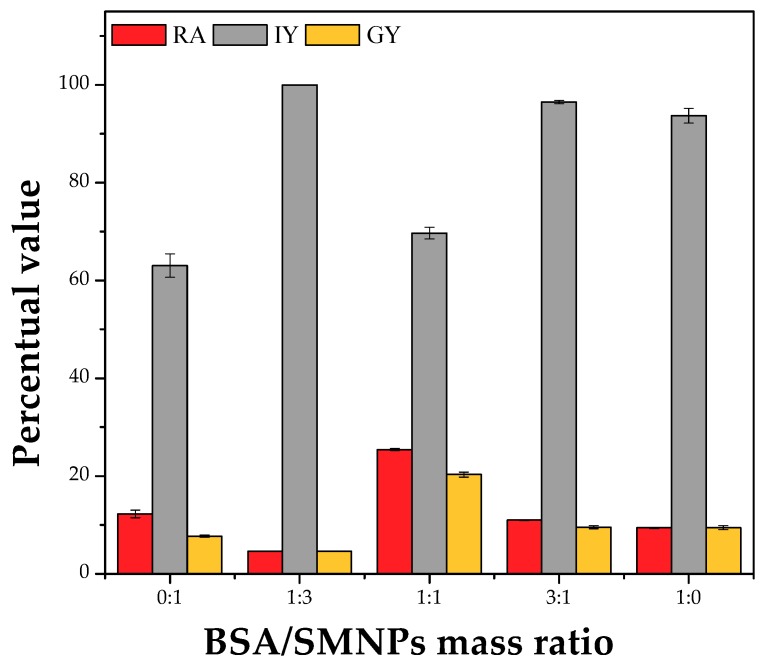
Effect of the BSA/SMNPs mass ratio on the immobilization parameters (RA—recovered activity, IY—immobilization yield, and GY—global yield) of porcine pancreas lipase (PPL) as CLEAs. Assay conditions: precipitation with ethanol (enzyme solution/ethanol volume ratio of 1:3), co-aggregation with additives (enzyme/total additives mass ratio of 1:3), crosslinking with glutaraldehyde (5 µmoles of glutaraldehyde/mg total protein), temperature of 4 °C, 30 min precipitation/aggregation and 2.5 h crosslinking under 150 rpm stirring.

**Figure 3:**
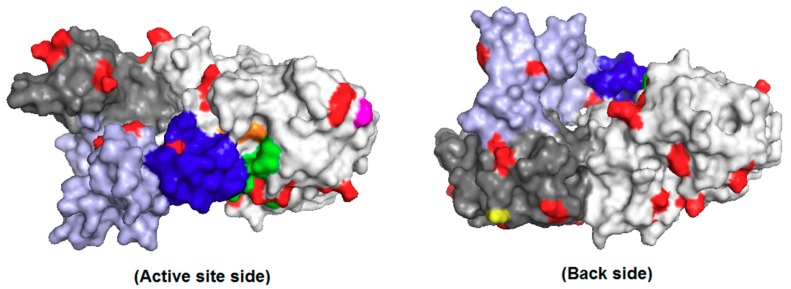
Three-dimensional structure of porcine pancreas lipase-colipase complex made with the program PyMol (The PyMol Molecular Graphics System; Version 2.1.0; Schrödinger, LLC). The complex (only chains A and B) is shown as surface mode from PDB structure (access code 1ETH). Chains A (*N*-terminal domain in light gray and *C*-terminal domain in dark gray) and B (colipase in light blue) are shown, and amino acid residues are highlighted in red (Lys), orange (Ser^153^, Asp^177^, and His^264^ catalytic triad), magenta (*N*-terminal), yellow (*C*-terminal), blue (lid–Cys^238^–Cys^262^ segment), and green (loop—residues 77–88).

As the combination of BSA and SMNPs (mass ratio of 1:1 to a total enzyme/BSA + SMMPs mass ratio of 1:3) did provide better results in terms of recovered activity and immobilization yield, other BSA/SMNPs mass ratios were also evaluated. Figure 2 shows that combinations of BSA/SMNPs with mass ratios of 1:3 and 3:1 yielded immobilization yields close to 100%; but decreasing the recovered activities below 10%. Perhaps the very rigid nanoparticles have a more negative effect on enzyme conformation when reacting with the enzyme, or their multifunctionality produced a somehow closer CLEA structure. PPL is a single polypeptide chain (~50 kDa) divided into two domains with specific functions (Figure 3). The *N*-terminal domain (residues from 1 to 336) contains the catalytic triad (Ser^153^, Asp^177^, and His^264^), and the *C*-terminal domain (residues from 337 to 449) is involved in the colipase binding, a small protein (~10 kDa) which anchors the lipase to the water-lipid interface [65]. This lipase presents an intrinsic flexibility in the relative position of their *N*- and *C*-terminal domains (a small rotation around residues 334–335). This property of the *N*-terminal domain to move independently from the lid (Cys^238^–Cys^262^ segment), colipase, and the *C*-terminal domain may have functional implications as the lipase molecule binds the water-lipid interface [65]. Besides, the lid and the loop comprising residues 77–86 (both critical to the lipase activation—opening the lid) contain a Lys residue (Lys^240^ and Lys^81^, respectively). The involvement of these Lys residues in covalent links with glutaraldehyde may negatively affect the enzyme activity.

Thus, the mass ratios enzyme/(BSA + SMNPs) of 1:3 and BSA/SMNPs of 1:1 were chosen for further set of experiments.

### 2.2. Treatment of PPL Surface

**Figure 4:**
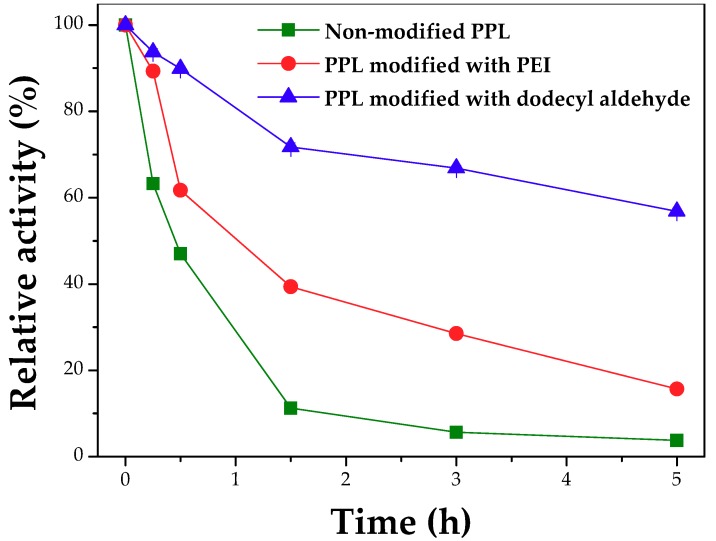
Thermal inactivation of free PPL non-modified and modified with polyethyleneimine (PEI) and dodecyl aldehyde. Assay conditions: 40 °C and pH 8.0 (100 mM phosphate buffer) and enzyme solutions containing 5 mg protein/mL.

The modification of the PPL surface with polyethyleneimine (PEI) and dodecyl aldehyde did lead to a loss of around 30% of the activity, where the specific activities (U/mg protein) were 27.54 ± 0.67, 19.44 ± 0.53 and 19.63 ± 0.18, for the unmodified enzyme, PEI or aldehyde treated enzyme, respectively. Table S1 in the Supplementary Materials shows that the modification reduces by approximately 30% the color obtained using TNBS titration. This means that around 344 µmol amino groups/g protein have been modified after this treatment. The surface of PPL is rich in primary amino groups (Figure 3). The chain A (*N*- and *C*-terminal domains) has 22 Lys residues and the amino terminal, while chain B (colipase) has 4 Lys residues (both number of Lys residues was obtained from the primary sequence of the lipase-colipase complex—PDB code 1ETH). Thus, particularly the low modification of amino groups by dodecyl aldehyde does not compromise the crosslinking of the enzyme with glutaraldehyde in the CLEA preparation because of the free primary amino groups still available in the enzyme surface. Despite that, the modified lipases were more stable than the non-modified LPP (Figure 4). Particularly, the PPL treated with dodecyl aldehyde showed much greater stability than the non-modified enzyme (residual activities after 5 h incubation at 40 °C and pH 8 (100 mM phosphate buffer) around 57% and 4%, respectively). This modification is under study in our research group.

Table 2 shows the effect of the treatment of the PPL surface with polyethyleneimine (PEI) and dodecyl aldehyde on the immobilization yield and recovered activity. All treatments allowed 100% immobilization yield, but the recovered activity of the biocatalysts did not show any improvement related to the CLEAs prepared with PPL without any treatment. 

The CLEAs prepared with PPL treated with PEI yielded the lowest recovered activity (7.17%), probably due to the formation of more compact structures promoted by the intense crosslinking. Besides, the cationic character of this polymer could form a hydrophilic layer on the enzyme surface, impairing substrate partition from the bulk to the active site. On the other hand, the treatment of PPL surface with dodecyl aldehyde (a hydrophobic aldehyde) allowed a recovered activity of around 20%, because this compound probably did created a hydrophobic layer on the enzyme surface, aiding in the aggregation step, even although the amino groups in the enzyme surface will be decreased for the crosslinking step. As the combination of both treatments did allow a small increase in the recovered activity, only the surface treatment with dodecyl aldehyde was chosen for further experiments.

### 2.3. Preparation of Magnetic CLEAs Using Starch in the Aggregation and Crosslinking Steps

**Figure 5:**
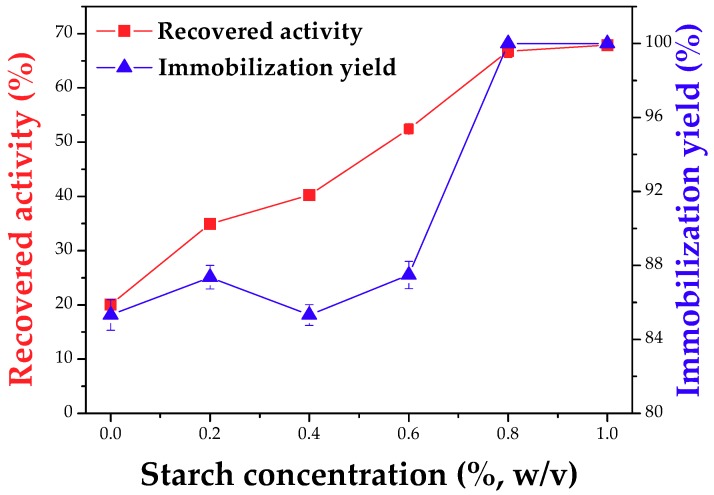
Effect of starch concentration on immobilization yield and recovered activity of PPL CLEAs. Assay conditions: treatment of PPL surface with dodecyl aldehyde (PPL/dodecyl aldehyde mass ratio of 1:1), precipitation with ethanol (enzyme solution/ethanol volume ratio of 1:3), co-aggregation with co-feeder (PPL/BSA/SMNPs mass ratio of 1:1.5:1.5 and starch concentration from 0.2 to 1%, *w*/*v*), crosslinking with glutaraldehyde (5 µmoles of glutaraldehyde/mg total protein), temperature of 4 °C, 30 min precipitation/aggregation and 2.5 h crosslinking stirred at 150 rpm and incubation with α-amylase at 25 °C stirred at 150 rpm for 2 h.

The influence of the starch concentration on the immobilization yield and recovered activity of CLEAs of PPL is shown in Figure 5. The addition of starch provided significant increases in the immobilization yield until a concentration of 0.6% (*w*/*v*), but only the concentrations of 0.8 and 1% (*w*/*v*) yielded 100% immobilization. The recovered activity increased for all starch concentrations, reaching maximum catalytic retention of 67% (more than doubling of the activity recovered without starch) when 0.8% starch was used during CLEA preparation. Thus, 0.8% (*w*/*v*) of starch was selected for the preparation of CLEAs of PPL.

Talekar et al. [12] reported the evaluation of starch on porous CLEAs (p-CLEAs) of invertase from *Saccharomyces cerevisiae* and found that the use of starch led to the formation of a non-uniform porous surface, resulting in better mass transfer and minimization of diffusion problems. Besides that, they concluded that 0.4% (*w*/*v*) starch concentration yielded 100% catalytic potential, demonstrating the feasibility of this enzyme aggregate preparation method.

### 2.4. Influence of Time of Glutaraldehyde Treatment in the Preparation of PPL CLEAs

The influence of the glutaraldehyde treatment time of the PPL CLEAs on the recovered activity was analyzed, and results pointed that this has not influence on the recovered activities, which were statistically equal by the Tukey test (around 66%). The incubation of the PPL CLEAs in phosphate buffer at high ionic strength for 12 h showed high stability against leaching (80 to 90% recovered activity). Despite this, 15 h crosslinking time was chosen for further assays [66].

### 2.5. Selection of Additives for Preparation of PPL CLEAs Using 0.8% (w/v) Starch as Pore-Making Agent

The influence of the presence of protein feeders (BSA, SP) and silica magnetic nanoparticles (SMNPs) in the preparation of PPL CLEAs using starch (0.8%, *w*/*v*) as pore-making agent is shown in Table 3. The recovered activities of all CLEAs prepared in the presence of starch increased more than twice after starch hydrolysis with α-amylase to wash away starch molecules, reaching 81% recovered activity when the PPL CLEA was prepared in the presence of a mix of SP and SMNPs. CLEAs prepared only with SMNPs were less stable when incubated at high ionic strength (59.8% recovered activity) than those prepared with BSA plus SMNPs and SP plus SMNPs (91% recovered activities). In view of these results, porous magnetic CLEAs (hereinafter abbreviated as pm-CLEA) prepared with SP plus SMNPs (pm-SP-CLEA) and BSA plus SMNPs (pm-BSA-CLEA) were selected to evaluate the effect of temperature, pH, agitation rate, and substrate concentration on the relative activity of this immobilized enzyme.

### 2.6. Characterization of Free and Immobilized PPL

#### Effect of pH, Temperature and Agitation Rate on Hydrolytic Activity and Thermal Stability

**Figure 6:**
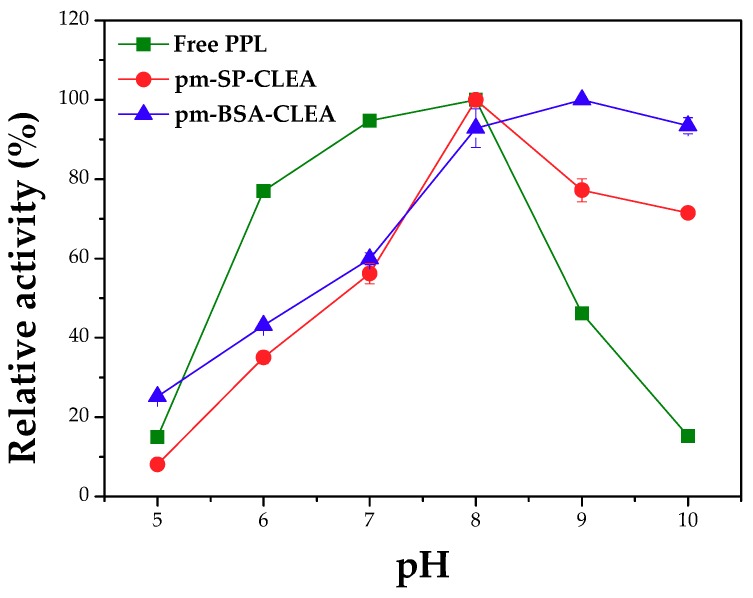
Profile of activity vs. pH for free and immobilized PPL. pm-(SP or BSA)-CLEA preparation conditions: treatment of PPL surface with dodecyl aldehyde (PPL/dodecyl aldehyde mass ratio of 1:1), aggregation/precipitation with ethanol (enzyme solution/ethanol volume ratio of 1:3) in presence of PPL/BSA/SMNPs or PPL/SP/SMNPs mass ratio of 1:1.5:1.5 and starch (0.8% *w*/*v*) for 0.5 h, followed by treatment with glutaraldehyde (5 µmoles of glutaraldehyde/mg total protein) for 15 h at 4 °C under 150 rpm stirring, and treatment with alpha-amylase at 25 °C under 150 rpm stirring for 2 h. Activity assay conditions: hydrolysis of tributyrin (217.9 mM concentration) at 40 °C and different pH values for 5 min under 500 rpm stirring.

The effect of pH on the kinetic profile of free and immobilized PPL (pm-CLEA) is shown in Figure 6. The pH values where maximum activities for free PPL (32.2 ± 0.65 U/mg protein) and pm-SP-CLEA (24.13 ± 0.35 U/mg protein) were found were similar (around pH 8.0), while for pm-BSA-CLEA (19.14 ± 0.13 U/mg protein) was shifted to 9.0. At higher pH values free enzyme rapidly decrease the activity, while the immobilized enzymes retained a high percentage of activity even at pH 10. That way, at alkaline conditions, pm-CLEAs were much more actives than the soluble counterpart in absolute terms. Cui et al. [45] also reported similar behavior for CLEAs of bovine pancreas lipase (above 90% activity in the pH range from 8.0 to 10.0).

**Figure 7:**
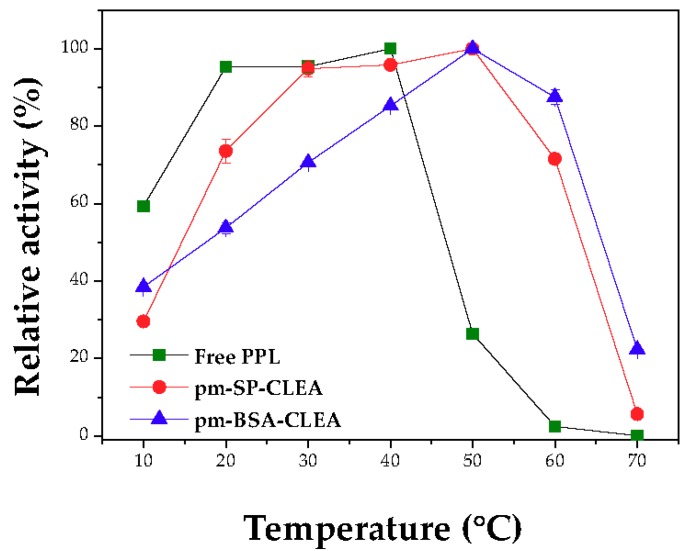
Activity profiles of PPL vs. temperature. pm-(BSA or SP)-CLEA preparation conditions: treatment of PPL surface with dodecyl aldehyde (PPL/dodecyl aldehyde mass ratio of 1:1), aggregation/precipitation with ethanol (enzyme solution/ethanol volume ratio of 1:3) in presence of PPL/BSA/SMNPs or PPL/SP/SMNPs mass ratio of 1:1.5:1.5 and starch (0.8% *w*/*v*) for 0.5 h, followed by treatments with glutaraldehyde (5 µmoles of glutaraldehyde/mg total protein) for 15 h at 4 °C under 150 rpm stirring, and with α-amylase at 25 °C under 150 rpm stirring for 2 h. Activity assay: hydrolysis of tributyrin solution pH 8.0 (217.9 mM concentration), temperature ranging from 10 to 70 °C for 5 min under 500 rpm stirring.

Figure 7 shows the effect of temperature on the activities of free and immobilized PPL. The temperature where the maximum activities of the porous magnetic CLEAs (pm-SP-CLEA and pm-BSA-CLEA) were detected was shifted from 40 °C (free PPL) to around 50 °C, suggesting that the immobilized enzyme is more thermally stable than the free PPL at high temperatures.

At 60 °C, pm-BSA-CLEA and pm-SP-CLEA presented specific activities of 28.0 ± 0.6 U/mg protein and 20.0 ± 0.8 U/mg protein, respectively, while free PPL showed an activity of 2.0 ± 0.1 U/mg protein. Again, at these conditions the immobilized enzyme becomes much more active than the free enzyme. Differences increase at higher temperature, e.g., at 70 °C the free enzyme is fully inactive while pm-BSA-CLEA retained more than 20% of the activity. These results show that the pm-BSA-CLEA presented greater stability in its structure under very drastic conditions than that of the free PPL since the immobilization produced a strong rigidification of the enzymatic structure.

Thus, the new biocatalyst presented better catalytic performance under drastic conditions than the free enzyme and is a new example on how the recovered activity may be altered depending on the activity determination conditions [22].

**Figure 8:**
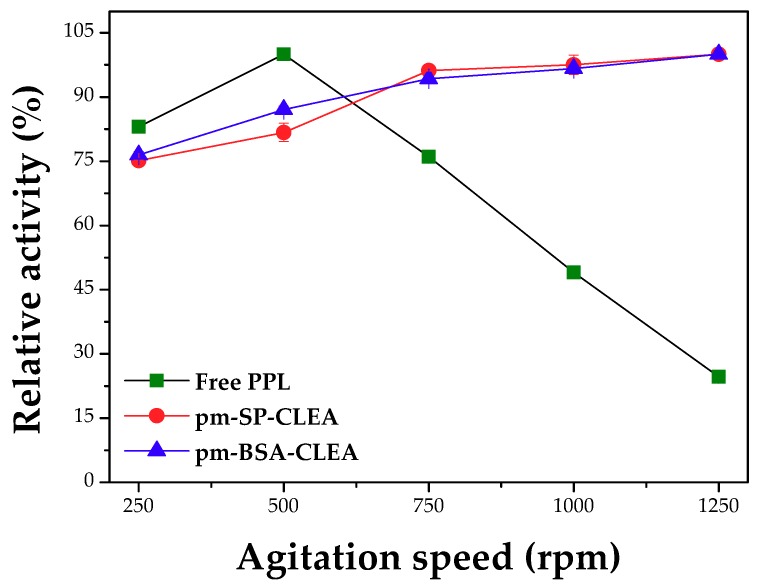
Effect of the agitation on the catalytic performance of free and immobilized PPL. Conditions of pm-(BSA or SP)-CLEA preparation: treatment of PPL surface with dodecyl aldehyde (PPL/dodecyl aldehyde mass ratio of 1:1), aggregation/precipitation with ethanol (enzyme solution/ethanol volume ratio of 1:3) in presence of PPL/BSA/SMNPs or PPL/SP/SMNPs mass ratio of 1:1.5:1.5 and starch (0.8% *w*/*v*) for 0.5 h, followed by treatments with glutaraldehyde (5 µmoles of glutaraldehyde/mg total protein) for 15 h at 4 °C under 150 rpm stirring, and with α-amylase at 25 °C under 150 rpm stirring for 2 h. Activity assay conditions: hydrolysis of tributyrin solution (217.9 mM concentration) at 40 °C and pH 8.0 for 5 min under different agitation velocities (250, 500, 750, 1000 and 1250 rpm).

Figure 8 shows the effect of the stirring rate on the kinetic behavior of the free and immobilized PPL. Free PPL showed maximum catalytic activity at 500 rpm and then, the activity decreased, while the activities of the porous magnetic CLEAs continuously increased within the interval evaluated. Thus, while the free enzyme is exposed to gas bubbles and this produces its inactivation, the immobilized enzyme is protected inside the solid porous particle and retained very high activity [19], and only the positive effects of the reduction of external mass transfer is observed.

**Figure 9:**
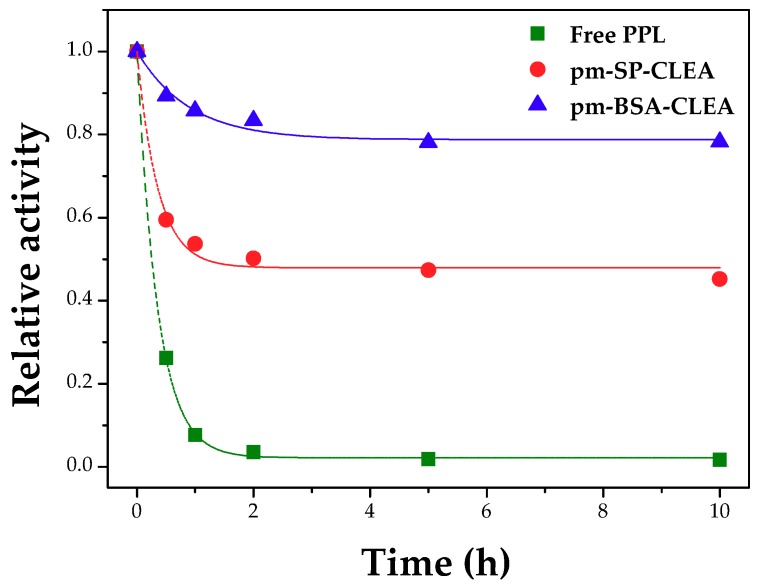
Thermal inactivation profiles of free and immobilized PPL at 40 °C and pH 8.0 under 150 rpm stirring. Biocatalyst preparation conditions: treatment of PPL surface with dodecyl aldehyde (PPL/dodecyl aldehyde mass ratio of 1:1), aggregation/precipitation with ethanol (enzyme solution/ethanol volume ratio of 1:3) in presence of PPL/BSA/SMNPs or PPL/SP/SMNPs mass ratio of 1:1.5:1.5 and starch (0.8% *w*/*v*) for 0.5 h, followed by treatments with glutaraldehyde (5 µmoles of glutaraldehyde/mg total protein), for 15 h at 4 °C under 150 rpm stirring, and with α-amylase at 25 °C for 2 h. Activity assay conditions: hydrolysis of 217.9 mM tributyrin solution at 40 °C and pH 8.0 for 5 min under 500 rpm stirring.

Figure 9 shows the inactivation profiles of free and immobilized PPL. It can be seen that pm-SP-CLEA exhibited high stability at 40 °C and pH 8.0, retaining around 80% of activity after 10 h incubation. The pm-BSA-CLEA was slightly less stable, but still retained around 50% of the initial activity after 10 h incubation, while free PPL was fully inactivated after 2 h incubation. The highest stabilities of the porous magnetic CLEAs are indicatives of efficient covalent cross-links stablished in the supramolecular structure of the CLEAs, turning the enzyme molecules more rigid and preventing distortional tridimensional changes at high temperatures. The biphasic profiles of the inactivation curves suggest that the porous magnetic CLEAs of PPL have a fraction of enzyme molecules more cross-linked and thereby more thermally stable. Perhaps this is the fraction of the PPL molecules directly attached to the nanoparticle, where stabilization effects should be higher counting by the higher rigidity of the nanoparticle compared to a protein [22].

### 2.7. Determination of Effectiveness Factors

**Figure 10:**
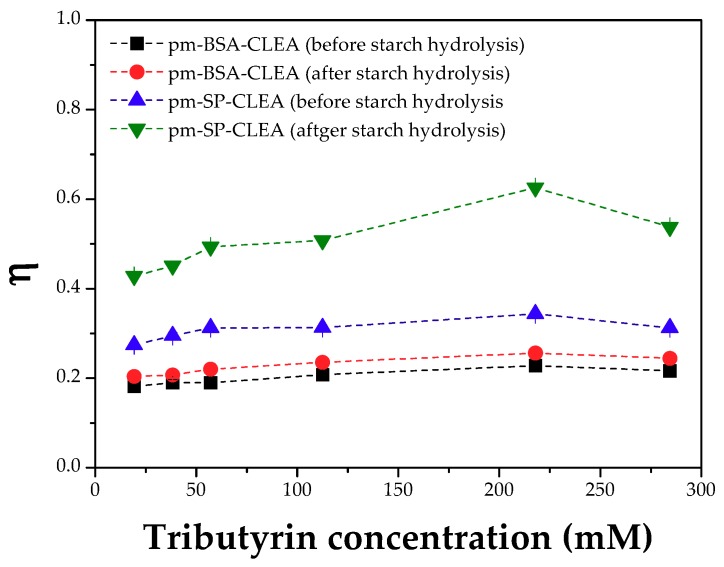
Effectiveness factors (η) for CLEAs of PPL. Biocatalyst preparation conditions: treatment of PPL surface with dodecyl aldehyde (PPL/dodecyl aldehyde mass ratio of 1:1), aggregation/precipitation with ethanol (enzyme solution/ethanol volume ratio of 1:3) in presence of LPP/BSA/SMNPs or LPP/SP/SMMPs mass ratio of 1:1.5:1.5 and starch (0.8% *w*/*v*) for 0.5 h, followed by treatment with glutaraldehyde (5 µmoles of glutaraldehyde/mg total protein) for 15 h at 4 °C under 150 rpm. Starch was washed away by hydrolysis with α-amylase at 25 °C for 2 h under 150 rpm stirring. Activity assay conditions: hydrolysis of tributyrin solutions (19.28, 38.35, 57.2, 112.5, 217.9 and 284.4 mM) at 40 °C and pH 8.0 for 5 min under 500 rpm stirring.

**Figure 11:**
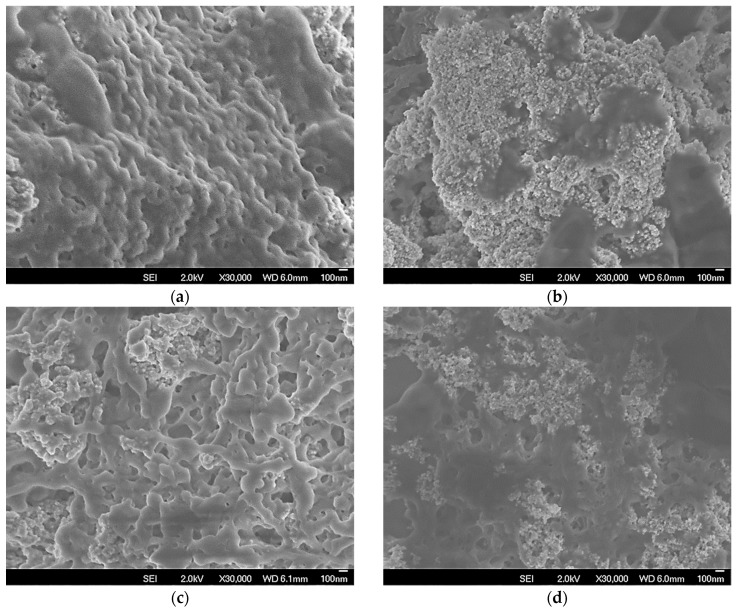
SEM-FEG images of pm-CLEAs of porcine pancreas lipase co-aggregated with (**a**) SMNPs and bovine serum albumin (pm-BSA-CLEA), (**b**) SMNPs and soy protein (pm-SP-CLEA), followed by treatment with α-amylase (**c**,**d**, respectively). All CLEAs were prepared under the same conditions, the only difference is if the starch (pore-making agent) was not hydrolyzed (**a**,**b**) or was hydrolyzed (**c**,**d**) with α-amylase.

CLEAs prepared in presence of starch as pore-making agent were evaluated as their effectiveness factors. Figure 10 shows that the evolution of the effectiveness factors (η = observed activity of CLEA/activity of an equivalent amount of free PPL) at growing concentrations of tributyrin. These factors were much higher after washing away the starch, mainly for CLEAs prepared in presence of soy protein and SMNPs. SEM-FEG images of CLEAs before starch hydrolysis (Figure 11) show a quite smooth and non-porous surface, which may result in serious mass transfer problems.

On the other hand, images of CLEAs after starch hydrolysis show non-uniform and porous surface due to removal of the starch molecules from the structure of the enzyme aggregate, which could explain the higher effectiveness factors (around η = 0.65), mainly for CLEAs prepared in presence of soy protein and SMNPs. On the other hand, PPL co-aggregated with BSA and SMNPs (pm-BSA-CLEAs) did not show significant increase in the observed activity, even using starch as pore-making agent. These results showed that the combination of SMNPs and soy protein as aids in the aggregation step and starch as pore-making agent was a good strategy to reduce mass transfer problems within the supramolecular structure of the CLEA.

### 2.8. Evaluation of Tributyrin Hydrolysis and Biocatalyst Reuse

**Figure 12:**
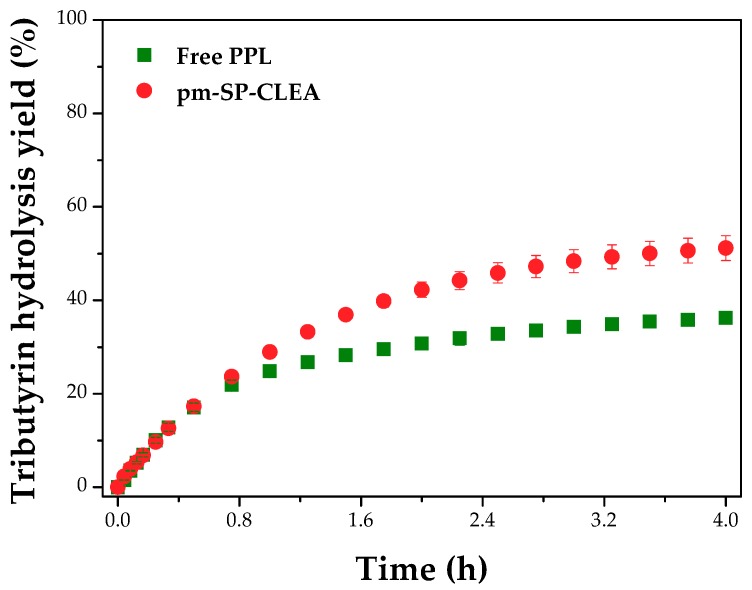
Yield of the hydrolysis of tributyrin with the reaction time at 37 °C and pH 7.5 stirred at 500 rpm catalyzed by free PPL and pm-SP-CLEA (74.22 mM tributyrin and 70 TBU/g tributyrin). Yield was calculated as percentage of butyric acid released (in μmoles) in relation to the total theoretical value.

Figure 12 shows the reaction course of the hydrolysis of tributyrin catalyzed by free PPL and pm-SP-CLEA. Figure 12 shows how the reaction catalyzed by free enzyme rapidly started to lose linearity, but this is not so obvious using the immobilized enzyme. The yields after 4 h of reaction were 36% and 52% using free PPL and pm-SP-CLEA, respectively. These results confirmed the lower stability of free PPL compared to the immobilized counterpart.

**Figure 13:**
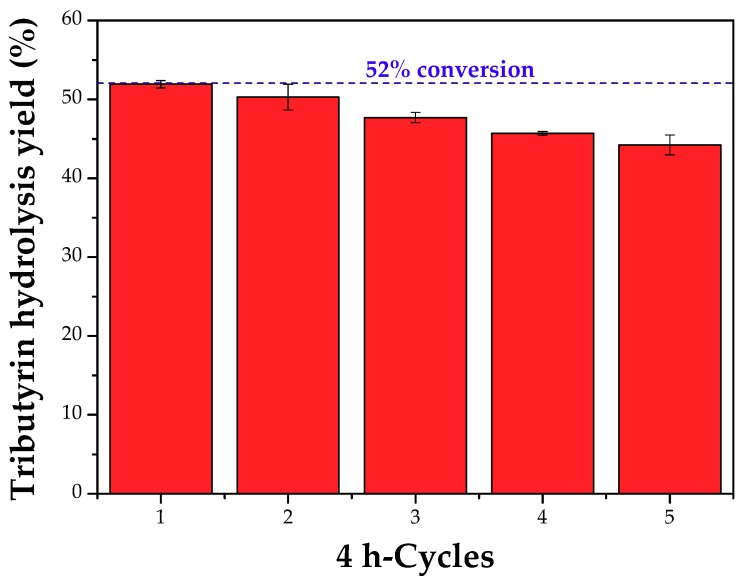
Reuse assay (4 h-batches) of the pm-SP-CLEA in the hydrolysis of tributyrin (74.22 mM) at 37 °C and pH 7.5 stirred at 500 rpm. Reaction medium: 6 mL sodium phosphate buffer (100 mM, pH 7.5), 16.5 mL distilled water, 0.5 mL tributyrin, and 70 TBU/g tributyrin.

Figure 13 shows that after five 4 h-batches, the yield of tributyrin hydrolysis decreased only 7% (from 52% to 45%). Besides the good operational mechanical stability, it is noticed that the recovery of the pm-SP-CLEA was very ease using a magnetic separation. 

It should be considered that free enzyme was quite unstable just at 3 °C more (Figure 9), while the immobilized enzyme can be reused for many cycles without significant changes. Moreover, di- and monobutyrin have some detergent effects that apparently have not very deleterious effects on enzyme stability even at 37 °C [67,68,69].

## 3. Materials and Methods

Lipase from porcine pancreas type II (PPL), bovine serum albumin (BSA), *tert*-butyl alcohol, tributyrin, polyethyleneimine (PEI, average M_n_ ~ 423), dodecyl aldehyde, and Bradford reagent were purchased from Sigma-Aldrich (St. Louis, MO, USA). Glutaraldehyde solution (25% in H_2_O) was purchased from Vetec Química Fina (Duque de Caixas, RJ, Brazil). Soluble starch was purchased from Panreac Química (Barcelona, Spain). Anhydrous ethanol (99.8% P.A.) was purchased from Synth (Diadema, SP, Brazil). Soy protein was acquired from local market. Silica magnetic nanoparticles (SMNPs) functionalized with amine groups derived from 3-aminopropyltriethoxysilane (APTS) (136 ± 10 µmol amino/g) were purchased from Kopp Technologies (São Carlos, SP, Brazil).

### 3.1. General Procedure of CLEA Preparation

In the precipitation step, ethanol (3.0 mL) was added to 1.0 mL of a homogeneous mixture containing PPL (5.0 mg of protein mL^−1^) and co-feeders (15 mg mL^−1^) prepared in 5.0 mM sodium phosphate buffer (pH 7.0). The mixture was maintained at 4 °C in an orbital shaker at 150 rpm stirring for 30 min. After this time, glutaraldehyde (5 μmoles of glutaraldehyde groups/mg total protein) was added and the crosslinking step proceeded for 2.5 h [12]. CLEAs prepared with protein co-feeders were separated by centrifugation at 10,400 g for 10 min at 4 °C, while CLEAs prepared with silica magnetic nanoparticles (SMNPs) as additive were separated by an external magnetic field. The precipitate (CLEAs of PPL) was washed twice with 3.0 mL of 100 mM phosphate buffer (pH 7.0), and finally resuspended in 1.0 mL of 5.0 mM phosphate buffer (pH 7.0). Measures of hydrolytic activities (tributyrin as substrate) in the initial enzyme solution, final supernatant, washing supernatants, and CLEA suspension were used to calculate the following immobilization parameters: immobilization yield (IY), recovered activity (RA), and global yield (GY) using the following equations:

(1) $$\mathrm{IY}=\frac{A_{i}-{(A}_{\mathrm{super}}+A_{\mathrm{washes}})}{A_{i}}\times 100$$

(2) $$\mathrm{RA}=\frac{A_{\mathrm{Derivative}}}{A_{i}-{(A}_{\mathrm{super}}+A_{\mathrm{washes}})}\times 100$$

(3) $$\mathrm{GY}=\frac{A_{\mathrm{derivative}}}{A_{i}}\times 100$$

where A_i_ is the initial activity; A_Derivative_ is the activity of the CLEAs, A_super_ is the activity of the supernatant and A_washes_ is the activity of the washing supernatants.

### 3.2. Preparation of Porous Magnetic CLEAs (pm-CLEAs)

Porous magnetic CLEAs (pm-CLEAs) were prepared by the general procedure described above but adding to the enzyme solution SMNPs and soluble starch as pore forming agent. In the precipitation step, 3.0 mL of ethanol were added to 1 mL of a homogeneous mixture containing PPL (5.0 mg of protein mL^−1^), protein feeder/additive (7.5 mg of BSA or soy protein and 7.5 mg of SMNPs), and soluble starch (0.2%, 0.4%, 0.6%, 0.8% and 1.0%, *w*/*v*) prepared in 5.0 mM sodium phosphate buffer (pH 7.0). The mixture was maintained at 4 °C in an orbital shaker stirred at 150 rpm for 30 min. After, glutaraldehyde (5 μmoles of glutaraldehyde/mg total protein) was added and the crosslinking step proceeded for 2.5 h. The precipitate was recovered by magnetic separation, washed, and resuspended in 3.0 mL of 5 mM phosphate buffer (pH 7.0). A volume of 100 μL α-amylase was added and the suspension was incubated at 25 °C for 2 h in order to hydrolyze starch into dextrins, maltose and glucose, which can be easily washed away [42]. After this time, the CLEAs were recovered by magnetic separation, resuspended in 1.0 mL of phosphate buffer (5.0 mM, pH 7.0), and stored at 4 °C. Hydrolytic activities were measured to calculate the immobilization parameters, as described above.

### 3.3. Protein Assay

Protein concentration was determined by Bradford’s method [70], using bovine serum albumin (BSA) as standard protein.

### 3.4. Standard Activity Assay

Hydrolytic activity was measured according to Beisson et al. [71] with minor modifications. Briefly, a volume of 100 μL of enzymatic solution (or resuspended CLEA) was added into a mixture of 1.5 mL of tributyrin, 6.0 mL of 100 mM sodium phosphate buffer (pH 7.5) and 16.5 mL of distilled water. The hydrolytic reaction was carried out at 37 °C, stirred at 500 rpm for 5 min. The tributyrin hydrolysis was monitored in a Titrino 907 titrator (Metrohm, Herisau, Switzerland) using a 20 mM KOH solution to keep the pH of reaction at 7.5. The hydrolytic activity was calculated considering the consumption of KOH to neutralize the butyric acid released in the reaction medium. One tributyrin unit (TBU) was defined as the amount of enzyme required to release 1 µmol of butyric acid per minute under the conditions described.

### 3.5. Chemical or Physical Modification of the Lipase Surface

In some instances, before the precipitation step, PPL was incubated with polyethyleneimine (PEI, oligomer mixture with an average Mn of 423) and/or dodecyl aldehyde.

The treatment with PEI was performed according to Wilson et al. [49], adding 50 μL of PEI solution (100 mg mL^−1^) in a homogeneous PPL solution (5 mg mL^−1^) prepared in 5 mM phosphate buffer (final pH 7.0). The reaction medium was incubated at 25 °C and stirred at 150 rpm for 60 min. At the end, the enzyme solution was dialyzed in a dialysis tubing cellulose membrane (typical molecular weight cut-off of 14 kDa) at 4 °C for 16 h against excess of water to remove excess of surface modifying agent.

For the treatment with dodecyl aldehyde, 181 μL of this aldehyde solution (831 mg mL^−1^) were added to 30 mL of a PPL solution (5 mg mL^−1^) prepared in 100 mM sodium carbonate buffer (pH 10.0) to give a dodecyl aldehyde:PPL mass ratio of 1:1. The solution was incubated at 25 °C under 150 rpm stirring for 180 min. In this case, both non-modified PPL and PPL treated with PEI were modified with dodecyl aldehyde. After, sodium borohydride (1 mg mL^-1^ solution) was added to the solution and the reaction proceeded for 30 min. At the end, the enzyme solution was dialyzed at 4 °C for 16 h against excess of water to remove excess of surface modifying and reducing agents.

The enzyme surface modification was evaluated by the colorimetric TNBS method [72]. Solutions of 0.1% (*v*/*v*) TNBS containing modified and non-modified PPL (0.01 mg mL^−1^) were prepared in 100 mM sodium borate pH 9.0 and incubated at 25 °C for 30 min. After, the absorbance was measured at 420 nm and it was related to amino group concentration using a standard curve constructed with glycine as standard amino acid (Figure S1).

### 3.6. Characterization of Free and Immobilized PPL

#### Effect of pH and Temperature on PPL Hydrolytic Activity and Thermal Stability

The enzymatic activity of immobilized or free enzyme was determined at different pH values and 37 °C, using 100 mM of different buffers: sodium acetate at pH 5.0, sodium phosphate at pH values from 6.0 to 8.0 or sodium carbonate at pH 9.0 and 10.0. A blank solution at same conditions (but without enzyme) was utilized to discount acid or alkaline chemical hydrolyses.

To determinate the optimum activity temperature of free or immobilized lipase, the enzymatic activity was measured using 100 mM sodium phosphate at pH 7.5 in a temperature range from 10 to 70 °C.

For stability assays, free and immobilized PPL were incubated at 40 °C and 100 mM phosphate buffer pH 8.0 for 10 h under 500 rpm stirring. At regular time intervals, samples were withdrawn for measurement of hydrolytic activity.

### 3.7. Determination of Effectiveness Factor

Initial reaction rates were measured at 40 °C and pH 7.5 using different tributyrin concentrations (19.28, 38.35, 57.2, 112.5, 217.9, and 284.4 mM) for free and immobilized PPL. Effectiveness factor (η) for each form of PPL and tributyrin concentration was determined using the following equation:

(4) $$\eta =\frac{V_{\mathrm{imm}}}{V_{\mathrm{free}}}$$

where, V_imm_ and V_free_ are the rates of the reaction catalyzed by the same amount of enzyme (free PPL and CLEAs of PPL, respectively).

### 3.8. Biocatalyst Reuse in Hydrolysis of Tributyrin

The performance of free PPL and pm-SP-CLEA was evaluated in the hydrolysis of a 74.2 mM tributyrin solution at 37 °C and pH 7.5 for 4 h under stirring at 500 rpm. The reaction medium was composed by 6 mL sodium phosphate buffer (100 mM, pH 7.5), 16.5 mL distilled water, 0.5 mL tributyrin, and an enzyme load of 70 TBU/g tributyrin (for free and immobilized PPL).

Reuse assays were carried out at the same conditions above using pm-SP-CLEAs as biocatalyst. Between each cycle, the CLEAs were recovered by magnetic separation and washed with distilled water.

### 3.9. Scanning Electron Microscopy with Field Emission Gun (SEM-FEG) of PPL CLEAs

The surface morphology of the CLEAs and pm-CLEAs was studied by scanning electron microscopy with field emission gun (SEM-FEG) using a JEOL JSM6701F (Germany) electron microscope operated at 2 kV. The samples were impregnated with silicon and dried in a desiccator for 24 h before being scanned under vacuum.

### 3.10. Statistical Analysis

All experiments were performed in triplicate. The results were expressed as an average ± standard deviation (σ). Analyses of variance between averages were performed by Tukey test at 5% significance.

## 4. Conclusions

The co-aggregation of porcine pancreas lipase (PPL) with protein feeder (mainly soy protein) and silica magnetic nanoparticles activated with amino groups, using starch as pore-making agent, yielded porous magnetic CLEAs (pm-CLEAs) of PPL with high retained activity (around 80% recovered activity) and high effectiveness factor (up to 60% of the equivalent free activity) due to their porous structures. The pm-CLEAs were high stable (around 80% of retained activity after 10 h at 40 °C and pH 8.0) and easily separated by an external magnetic field, thus avoiding the formation of large clusters conventionally observed by centrifugation separation, which can aggravate mass transfer problems in the compacted CLEA structures. Besides, porous magnetic CLEAs co-aggregated with soy protein and magnetic nanoparticles (pm-SP-CLEAs) showed good performance and reusability in the hydrolysis of tributyrin for five 4h-batches. The use of magnetic nanoparticles can permit to immobilize the enzyme on its surface providing some extra-rigidification via multipoint covalent attachment [73], also may produce some order in the immobilized molecules that can provide some advantages [74].

## Figures and Tables

**Table 1:** Evaluation of the addition of silica magnetic animated nanoparticles (SMNPs) and different protein co-feeders in the preparation of CLEAs of porcine pancreas lipase (PPL). The values are expressed as average of triplicates ± standard deviation (σ).

CLEAs of PPL	RA ^a^ ± σ (%)	IY ^b^ ± σ (%)	GY ^c^ ± σ (%)
PPL CLEA ^d^	1.46 ± 0.08	65.25 ± 3.52	0.93 ± 0.003
PPL BSA-CLEA ^e^	9.40 ± 0.11	93.69 ± 1.50	9.45 ± 0.39
PPL SP-CLEA ^f^	22.81 ± 1.16	51.42 ± 0.35	10.72 ± 0.34
PPL SMNPs-CLEA ^g^	12.27 ± 0.77	63.04 ± 2.43	7.72 ± 0.24
PPL BSA-SMNPs-CLEA ^h^	25.43 ± 0.21	69.69 ± 1.2	20.30 ± 0.52
PPL SP-SMNPs-CLEA ^i^	32.84 ± 1.76	45.25 ± 1.07	16.40 ± 1.12

^a^ Recovered activity; ^b^ Immobilization yield; ^c^ Global yield; CLEA prepared ^d^ without co-feeders and with: ^e^ Bovine serum albumin (BSA); ^f^ Soy protein (SP); ^g^ Silica magnetic nanoparticles (SMNPs); ^h^ BSA/SMNPs mass ratio of 1:1; ^i^ SP/SMNPs mass ratio of 1:1. Assay conditions: precipitation with ethanol (enzyme solution/ethanol volume ratio of 1:3) and co-aggregation without and with co-feeders (enzyme protein/co-feeder mass ratio of 1:3) for 30 min, followed by crosslinking with glutaraldehyde (5 µmoles of glutaraldehyde/mg total protein) for 2.5 h at 4 °C under 150 rpm stirring.

**Table 2:** Evaluation of the treatment of porcine pancreas lipase (PPL) with polyethyleneimine (PEI) and dodecyl aldehyde on the immobilization parameters IY (immobilization yield) and RA (recovered activity). The values are expressed as mean of triplicates ± standard deviation (σ).

PPL Treatment with	RA ± σ (%)	IY ± σ (%)
PEI	7.17 ± 0.32	99.50 ± 1.00
Dodecyl aldehyde	20.08 ± 0.72	99.00 ± 2.00
PEI followed by Dodecyl aldehyde	25.64 ± 1.37	99.60 ± 2.00

Note: CLEA preparation conditions: treatment of PPL surface with PEI and dodecyl aldehyde, precipitation with ethanol (enzyme solution/ethanol volume ratio of 1:3), co-aggregation with co-feeder (PPL/BSA/SMNPs mass ratio of 1:1.5:1.5), crosslinking with glutaraldehyde (5 µmoles of glutaraldehyde/mg total protein), temperature of 4 °C, 30 min precipitation/aggregation and 2.5 h crosslinking under 150 rpm stirring.

**Table 3:** Evaluation of different additives in the preparation of porous magnetic CLEAs (pm-CLEAs) of porcine pancreas lipase (PPL). The values of the immobilization parameter RA (recovered activity) are expressed as average of triplicates ± standard deviation (σ).

Additives	Recovered Activities of Magnetic CLEAs (RA ± σ (%))	
	Before Starch Hydroysis with α-Amylase	After Starch Hydrolysis with α-Amylase
SMNPs ^a^	24.46 ± 0.24	54.01 ± 0.64
BSA + SMNPs ^b^	28.18 ± 0.67	66.55 ± 0.01
SP + SMNPs ^c^	36.77 ± 0.38	81.02 ± 0.16

^a^ Silica magnetic nanoparticles; ^b^ Bovine serum albumin/silica magnetic nanoparticles mass ratio of 1:1; ^c^ Soy protein/silica magnetic nanoparticles mass ratio of 1:1. Assay conditions: treatment of PPL surface with dodecyl aldehyde (PPL/dodecyl aldehyde mass ratio of 1:1), aggregation/precipitation with ethanol (enzyme solution/ethanol volume ratio of 1:3) in presence of additives (PPL/total additives mass ratio of 1:3) and starch (0.8%, *w*/*v*) as pore-making agent for 0.5 h at 4 °C under 150 rpm stirring, followed by treatment with glutaraldehyde (5 µmoles of glutaraldehyde/mg total protein) for 15 h. At the end, starch was hydrolyzed with α-amylase at 25 °C for 2 h under 150 rpm.

## Supplementary Materials

The following are available online at http://www.mdpi.com/1420-3049/23/11/2993/s1.
