# Supplementary material for: Evaluation of Strategies to Produce Highly Porous Cross-Linked Aggregates of Porcine Pancreas Lipase with Magnetic Properties
Source: Molecules. 2018 Nov 16;23(11):2993. doi: 10.3390/molecules23112993 (PMC6278321; doi:10.3390/molecules23112993)
Supplement: Supplementary file 1 [file molecules-23-02993-s001.pdf]

Supplementary Materials
Evaluation of strategies to produce highly porous cross-linked aggregates of porcine pancreas lipase with magnetic properties

José Renato Guimarães[1], Raquel de Lima Camargo Giordano[1], Roberto Fernandez-Lafuente[2*], Paulo Waldir Tardioli[1*]

[1] Graduate Program in Chemical Engineering, Department of Chemical Engineering, Federal University of São Carlos, Rod. Washington Luiz, São Carlos 13565-905, Brazil; renatoge74@gmail.com (J. R. G.); raquel@ufscar.br (R. L. C. G.); pwtardioli@ufscar.br (P.W.T).

[2] Departmento de Biocatálisis, ICP-CSIC, Campus UAM-CSIC Madrid, Madrid 28049, Spain; rfl@icp.csic.es (R. F.-L.);

* Correspondence: pwtardioli@ufscar.br (P.W.T); rfl@icp.csic.es (R.F.L); Tel.:+55-16-33519362 (P.W.T.); Tel:+34 915954941 (R.F.-L.).

Table S1. Evaluation of the treatment of porcine pancreas lipase (PPL) with polyethyleneimine (PEI) and dodecyl aldehyde by the 2,4,6-Trinitrobenzenesulfonic Acid (TNBS) colorimetric method for the determination of amine groups.

PPL treatment with	Abs 420nm ± σ	[Amino groups] ± σ (μmol amino groups/g protein)
None	0.821 ± 0.068	1102.01 ± 90.611
PEI	0.981 ± 0.069	1316.51 ± 92.31
Dodecyl aldehyde	0.565 ± 0.012	757.85 ± 15.91

Assay conditions: (i) treatment with PEI: 50 μL of PEI solution (100 mg mL^{-1}) were added to 1 mL of a PPL solution (5 mg mL^{-1}) prepared in phosphate buffer (5 mM, final pH 7.0), followed by incubation at 25 °C for 60 min stirred at 150 rpm. (ii) treatment with dodecyl aldehyde: 181 μL of dodecyl aldehyde solution (831 mg mL^{-1}) were added to 30 mL of a PPL solution (5 mg mL^{-1}) prepared in sodium carbonate buffer (100 mM, pH 10.0), followed by incubation at 25 °C for 180 min stirred at 150 rpm. After each treatment, the enzyme solution was dialyzed against excess of distilled water in a dialysis tubing cellulose membrane (14 kDa cut-off) at 4 °C for 16 h. After dialysis, the enzyme surface modification was evaluated by the colorimetric TNBS method: solutions of 0.1% (v/v) TNBS containing modified and non-modified PPL (0.01 mg mL^{-1}) were prepared in 100 mM sodium borate pH 9.0 and incubated at 25 °C for 30 min. After, the absorbance was measured at 420 nm and it was related to amino group concentration using a standard curve constructed with glycine as standard amino acid.

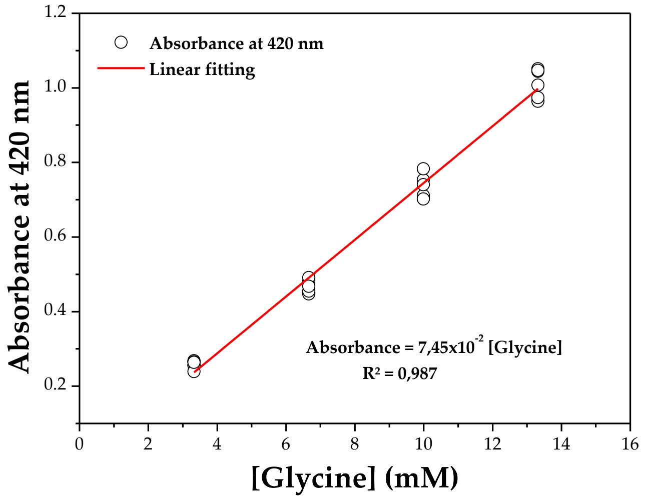

Figure S1. Standard curve "absorbance vs. concentration of amino groups" using glycine as standard amino acid. Assay conditions: solutions of 0.1% (v/v) TNBS containing glycine (0.25, 0.5, 0.75 and 1 mg mL^{-1}) were prepared in 100 mM sodium borate pH 9.0 and incubated at 25 °C for 30 min. After, absorbance was measured at 420 nm. The concentration of amine groups was determined by the stoichiometric relation of glycine and amine groups present in this amino acid.
